# Ethical Alternatives to Experiments with Novel Potential Pandemic Pathogens

**DOI:** 10.1371/journal.pmed.1001646

**Published:** 2014-05-20

**Authors:** Marc Lipsitch, Alison P. Galvani

**Affiliations:** 1 Center for Communicable Disease Dynamics, Department of Epidemiology, Harvard School of Public Health, Boston, Massachusetts, United States of America; 2 Department of Immunology and Infectious Diseases, Harvard School of Public Health, Boston, Massachusetts, United States of America; 3 Department of Epidemiology (Microbial Diseases), Yale School of Public Health, New Haven, Connecticut, United States of America; 4 Department of Ecology and Evolutionary Biology, Yale School of Public Health, New Haven, Connecticut, United States of America

## Abstract

*Please see later in the article for the Editors' Summary*

**Summary Points**

- “Gain of function” experiments involving the creation and manipulation of novel potential pandemic pathogens (PPPs) deserve ethical scrutiny regarding the acceptability of the risks of accidental or deliberate release and global spread.
- The Nuremberg Code, a seminal statement of clinical research ethics, mandates that experiments that pose a risk to human life should be undertaken only if they provide humanitarian benefits that sufficiently offset the risks and if these benefits are unachievable by safer means.
- A novel PPP research program of moderate size would pose substantial risks to human life, even optimistically assuming a low probability that a pandemic would ensue from a laboratory accident.
- Alternative approaches would not only be safer but would also be more effective at improving surveillance and vaccine design, the two purported benefits of gain-of-function experiments to create novel, mammalian-transmissible influenza strains.
- A rigorous, quantitative, impartial risk–benefit assessment should precede further novel PPP experimentation. In the case of influenza, we anticipate that such a risk assessment will show that the risks are unjustifiable. Given the risk of a global pandemic posed by such experiments, this risk assessment should be part of a broader international discussion involving multiple stakeholders and not dominated by those with an interest in performing or funding such research.

Two recent publications reporting the creation of ferret-transmissible influenza A/H5N1 viruses [1],[2] are controversial examples of research that aims to produce, sequence and characterize “potential pandemic pathogens” (PPPs) [3], novel infectious agents with known or likely efficient transmission among humans, with significant virulence, and for which there is limited population immunity. There is a quantifiable possibility that these novel pathogens could be accidentally or deliberately released. Exacerbating the immunological vulnerability of human populations to PPPs is the potential for rapid global dissemination via ever-increasing human mobility. The dangers are not just hypothetical. The H1N1 influenza strain responsible for significant morbidity and mortality around the world from 1977 to 2009 is thought to have originated from a laboratory accident [4].

Risk evaluations surrounding biomedical research have not kept pace with scientific innovations in methodology and application. This gap is particularly disconcerting when research involves the construction of PPPs that pose risks of accidental release and global spread. We argue here that accepted principles of biomedical research ethics present a high bar to PPP experiments, requiring that risks arising from such experiments be compensated by benefits to public health not achievable by safer approaches. Focusing on influenza, the object of most current PPP experimentation, we further argue that there are safer experimental approaches that are both more scientifically informative and more straightforward to translate into improved public health through enhanced surveillance, prevention, and treatment of influenza.

## Influenza “Gain of Function” Experiments: Prototypical Examples of Potential Pandemic Pathogen Studies

Although several pathogens may be categorized as PPPs (see Box 1), “gain of function” experiments involving influenza strains modified to be PPPs are expanding [5]–[7] (Box 2), and hence of immediate concern. In addition to the two controversial studies recently published, studies with H5N1 [8], H7N9 [9], and H7N1 [10] have used similar ferret passage protocols, while still others have created mammalian-transmissible strains in vitro, followed by in vivo analysis [11],[12]. Related studies have genetically combined less pathogenic zoonotic avian viruses, such as H9N2, with human seasonal influenza viruses to generate strains that exhibit enhanced transmissibility, and to which humans would be immunologically susceptible [13]–[15].

**Box 1. Scope for Heightened Ethical Scrutiny of Potential Pandemic Pathogen Experiments**

This article describes the responsible ethical scrutiny that should be applied to experimental studies creating or employing PPPs. We define PPPs as infectious agents with four characteristics:

1. Having known or likely efficient transmission among humans
2. Significantly virulent
3. Unmitigated by preexisting population immunity
4. Genetically distinct from pathogens currently circulating

These criteria define pathogens on which experimentation would pose a risk of sparking a pandemic, placing the human population at risk of morbidity or mortality, over and above the background risk of a naturally occurring pandemic. The paradigm case is the creation of variants of influenza A/H5N1 that are readily transmissible between ferrets, a model for human transmission. Such criteria would likely be applicable to experimentation with human isolates of smallpox or SARS, since these pathogens are no longer known to be circulating naturally. In the future, the list may expand [57].

We do not advocate the necessity of heightened scrutiny for isolation and characterization of naturally occurring pathogens, such as wild-type H5N1 or H7N9, consistent with the HHS framework for evaluating gain-of-function studies of H5N1 viruses, which exempts characterization of naturally occurring viruses [33].

**Box 2. Gain of Function: What's in a Name?**

The recent ferret transmission experiments with influenza A viruses have been termed “gain of function” experiments because they involve engineering viruses that gain transmissibility in ferrets. Gain of function is a common and important approach in biological experimentation, and is not by itself cause for concern [58]. However, the elevated concern over these experiments arises from the particular function that is gained. Ferret transmission is thought to be a good [59],[60] (albeit imperfect [61],[62]) model for human-to-human transmission. Consequently, strains resulting from selection for heightened ferret transmission are likely to be similarly transmissible by humans via respiratory droplets, a prerequisite for pandemic spread. In combination with demonstrated virulence for humans, this particular gain of function presents unique risks and raises special ethical issues.

These studies have typically been conducted in biosafety level (BSL) 3 or 3+ containment facilities. Laboratory-associated infections in BSL3 facilities are conservatively estimated to occur at a rate of two per 1,000 laboratory-years [3],[16] in the United States, where protocols and enforcement are relatively stringent. Globally, high-containment laboratories have variable standards and enforcement [17]. Experimentation in less-regulated or unregulated laboratories, with the attendant risks of accidental or deliberate release, is facilitated by the publication of sequence and functional data on PPPs, even if the original research was conducted with state-of-the-art safety and security [18].

From the conservative estimate of the rate of laboratory-associated infections of two per 1,000 laboratory-years [3],[16], it follows that a moderate research program of ten laboratories at US BSL3 standards for a decade would run a nearly 20% risk of resulting in at least one laboratory-acquired infection, which, in turn, may initiate a chain of transmission. The probability that a laboratory-acquired influenza infection would lead to extensive spread has been estimated to be at least 10% [19]. Simple branching process models suggest a probability of an outbreak arising from an accidental influenza infection in the range of 5% to 60% [20],[21]. Such probabilities cannot be ignored when multiplied by the potential devastation of an influenza pandemic [22],[23], even if the resulting strain were substantially attenuated from the observed virulence of highly pathogenic influenza A/H5N1 [24], the subject of much of the published PPP work to date. We advocate a dispassionate review of pertinent evidence and calculations of the probabilities and magnitudes of potential risks parameterized for specific PPP research programs.

## Ethical Frameworks for Novel Potential Pandemic Pathogens

Thus far, experiments with novel PPPs have been assessed in the context of “dual use research of concern” (DURC), a designation for “research that could be used for good or bad purposes” [25]. Within the broader category of DURC, PPP experimentation raises ethical issues that deserve more extensive evaluation than other DURC, because the scale of risk posed by PPPs is much greater. While DURC by definition presents a risk of malevolent use, the impact of the *accidental* release of many agents involved in DURC—anthrax, hemorrhagic fever viruses, and, most recently, a novel *Clostridium botulinum* toxin [26],[27] –is constrained by transmission mode or limited host susceptibility. The magnitude of accidental risk for a novel PPP is much greater.

The Nuremberg Code, a seminal document in clinical research ethics, specifies that in research conducted on human participants, “the degree of risk to be taken should never exceed that determined by the humanitarian importance of the problem to be solved by the experiment.” More broadly, 74 national academies of science have stated: “Scientists have an obligation to do no harm. They should always take into consideration the reasonably foreseeable consequences of their own activities” [28]. The ethical principles underlying both guidelines would seem to apply a fortiori to research that imposes far-reaching risk to the public [29]. Given the global nature of influenza transmission, and thus implications beyond a country's borders, international agreement regarding acceptable risks is needed.

Ethical constructs and risk evaluations must be tailored to scientific advances in methodology and application. Limited attention has been paid to the ethics of scientific experiments that pose risks beyond identified human participants [30]–[32]. On a practical level, however, the spirit of the Nuremberg Code's “humanitarian importance” criterion is embodied in the recent frameworks for evaluating PPP experiments from the US Department of Health and Human Services (HHS), the primary sponsor of such experiments to date. The HHS frameworks for studies anticipated to create mammalian-transmissible H5N1 [33] and H7N9 [34],[35] viruses specify that the risks and benefits should be weighed.

The Nuremberg Code's second point states: “The experiment should be such as to yield fruitful results for the good of society, unprocurable by other methods or means of study, and not random and unnecessary in nature.” When projecting the benefits of experiments that put human life at risk, therefore, it is critical to compare against alternatives. What *unique* public health benefits do PPP experiments offer relative to the benefits of investing equivalent resources in alternative research strategies? If there are unique benefits to novel PPP experiments, do they justify the risks entailed? This concept, too, is partially incorporated in the HHS frameworks, which permit funding of H5N1 [33] or H7N9 [34],[35] transmissibility gain-of-function experiments only if “there are no feasible alternative methods to address the same scientific question in a manner that poses less risk than does the proposed approach” [33]. The Nuremberg Code suggests a broader criterion: that PPP experiments should be performed if the *public health* benefits envisaged cannot be obtained by safer methods. We argue that alternative scientific approaches are not only less risky, but also more likely to generate results that can be readily translated into public health benefits.

## Challenges in Translating Understanding of Influenza Transmission into Public Health Practice

Proponents of PPP experimentation cite two main benefits of such studies: improving our interpretation of surveillance data to detect dangerous viruses and facilitating vaccine development against future natural pandemics. Both claims have been disputed. The vaccine claim has been denied by vaccine developers, who note that many, if not all, vaccines have been developed without a detailed molecular understanding of transmission [36]. Advocates of PPP experimentation further argue that creating potentially pandemic strains of a particular virus, e.g., A/H5N1, could facilitate the production and stockpiling of vaccines against that variant. However, given that PPP experiments inevitably consider only a few possible genetic pathways to transmissibility, and that the precise correspondence between transmissibility in the ferret model and human transmissibility remains uncertain, we can never know whether PPP experimentation would hit upon the antigenic composition of the next pandemic strain that will emerge from nature. Indeed, as described below, it is clear that there is no one-to-one mapping between a few genetic changes in a virus and its transmissibility. By contrast, universal influenza vaccines currently in preclinical and clinical trials [37] may, with further development, prove to be more worthwhile to stockpile for the purposes of pandemic preparedness than an assortment of vaccines targeting antigenic variants manufactured via PPP experimentation.

Current surveillance is likely inadequate to detect an emerging pandemic strain before it is too late [29],[38], regardless of any warnings that PPP experimentation might generate about potentially worrisome mutations. Between 2008 and 2013, over 1,580 highly pathogenic avian influenza (almost all H5N1) outbreaks, involving over 5 million birds, were reported to the World Organisation for Animal Health [39]. The US National Center for Biotechnology Information Influenza Virus Resource [40] received about 1,400 complete or partial avian H5N1 virus sequences over this period [41]. Most of these sequences were over eight months old by the time they were publicly available in the Influenza Virus Resource. Similar considerations apply to GISAID's EpiFlu Database, the other major influenza virus sequence database [42]. Given that birds [43], like humans [44], harbor a genetically diverse quasispecies of influenza variants, it is highly unlikely that such limited surveillance could detect a pandemic viral sequence and, furthermore, spur effective mitigation actions, before the worrisome variant was already widespread in birds. As an example of the limited public health response even when a dangerous virus has been observed, consider the global response to H7N9 avian influenza, which has proven zoonotic potential and has probably been repeatedly transmitted from human to human [45]. Isolates from human cases reveal efficient binding to human sialic acid receptors and airborne transmission in ferrets [9] and guinea pigs [46]. These indicators of pandemic potential are much stronger than sequence comparisons with engineered viruses could provide, yet most live bird markets in China remain open, and human cases continue to emerge [47]. Given these realities, it is difficult to envision how a surveillance signal alone would prompt swifter actions than these existing warning signs for H7N9 have. In short, the benefits for public health of the scientific findings from PPP experimentation are speculative at best.

## Epistatic Interactions in Influenza Genetics: A Challenge to Predicting Complex Phenotypes

A further challenge to realizing public health benefits from PPP experimentation is that the predictability of phenotype from viral sequence is complex [38],[48],[49], as demonstrated by a recent assessment [50] of the generality of mutations that conferred human receptor binding in engineered ferret-transmissible H5N1 strains. When these mutations were introduced into the genetic background of more recent avian isolates of H5N1, affinity to human receptors was lost [50]. Instead, the phenotype of any mutation depends on interactions with its genetic background, a phenomenon known as epistasis that is observed broadly in nature and in influenza viruses specifically [51],[52] (Table 1). Thus, it is unlikely that a catalog of mutations could inform reliable predictions of transmission phenotype in circulating strains.

**Table 1:** Evidence of strong epistasis: examples of mutations in influenza A viruses and their varying phenotypes that are dependent on genetic background.

Mutation	Predicted Phenotype (Background)	Other Phenotype (Background)
PB2 E627K	Elevated virulence, transmissibility (H5N1, H1N1, H7N7, etc.) [63]–[66]	Unaffected virulence or transmissibility (H1N1pdm) [67]; decreased fitness (older H5N1) [66]
NA H275Y	Oseltamivir resistance, fitness crippled (H1N1) [68],[69]	Oseltamivir resistance, fitness increased in absence of drug (H1N1) [68],[70]
HA LS, 158,224,226	Mammalian transmission (H5N1 from Indonesia and Viet Nam) [1],[2]	No switch to mammalian sialic acid binding (H5N1 from Egypt) [50]
Polybasic HA cleavage site	High avian pathogenicity (many H5 and H7 viruses) [71],[72]	Low avian pathogenicity (four H5 isolates) [73]

HA, hemagglutinin; NA, neuraminidase.

## Alternative Approaches: Safer and More Promising

The prominent role of epistasis in influenza biology suggests that alternative approaches to studying the phenotypic impact of mutations on mammalian transmissibility would be not only less risky, but also more informative. In vivo replication and transmission of influenza in humans depend on myriad interdependent factors, including the binding affinity between hemagglutinin and human sialic acids, the ability of the virion to fuse with the endosomal membrane at the appropriate pH and temperature, as well as the stability of various viral proteins [11],[51],[53],[54]. Each of these traits, in turn, is not simply determined by the presence or absence of individual amino acids at particular sites, but by biophysical properties arising from the interaction of many sites within and between proteins [50],[51]. Consequently, the challenge of predicting transmissibility hinges on understanding the genetic determinants of each trait, coupled with the interactions of the traits from which the higher-level phenotype of transmissibility arises. An array of safer approaches (Table 2) to studying influenza pathogenesis and transmission focus on dissecting these interactions. Some approaches start with sequence analysis and molecular dynamics modeling, which are intrinsically safe. The experimental evaluation of hypotheses raised by such studies may use viral components rather than the entire infectious virus, making these experiments simultaneously safer and more precise and mechanistic than engineering PPPs. Furthermore, these approaches are typically less costly than PPP experimentation, facilitating phenotypic evaluation of a greater diversity and abundance of genetic variants. Ultimately, studies with intact viruses will be necessary for a full understanding of human transmissibility, a phenotype of a whole virus. Elucidating the evolutionary trajectory through which existing seasonal (former pandemic) viruses became transmissible from avian precursors is safer than PPP experimentation, given that there is preexisting population immunity to seasonal strains, the products of such evolution.

**Table 2:** Safer approaches to studying human adaptation of influenza A viruses, and more generally to improving vaccines and therapeutics.

Approach	Examples	Scientific Benefits
Molecular dynamical modeling of influenza proteins and interactions with inhibitors and receptors	Analysis of adaptive changes in HA of H1N1pdm [74], lipid tail protrusion as a determinant of HA–membrane fusion [75], and identifying determinants of inhibitor-resistant NA [76]	Biophysical basis for complex phenotypes
In vitro studies of specific properties required for human adaptation, using single proteins	Studies of H5 or H7 receptor binding to mammalian versus human sialic acids [50],[77]; studies of genetic determinants of optimal pH of fusion by comparing properties of natural isolates [11]	Higher throughput than in vivo studies; can study more sequences and define motifs required for binding, beyond individual mutations; ability to assess generality of hypothesized determinants [54]
In vitro studies of genetic interactions between loci in one or several viral proteins using replication-incompetent viruses	Studies of epistatic interactions in nucleoprotein [51] or between nucleoprotein and polymerase [78] based on in vitro expression of markers and stability measurements of proteins	Higher throughput; ability to link structure to function; ability to test combinations of mutations
Sequence database comparisons of genetic properties of human- and avian-adapted viruses	Identify amino acid markers of host adaptation and quantify the extent of adaptation to a particular host [79],[80]; search for markers of human adaptation (established from earlier studies without PPP production) in H7N9 viruses [63]	Very high throughput; future studies could use novel analytic methods [81] to systematically identify new markers associated with human adaptation, which could then be tested experimentally; focus on naturally viable mutations
Sequence and in vitro phenotypic comparisons of human seasonal influenza isolates, zoonotic isolates from infected humans, and avian isolates	Comparison of human and avian isolates of H7N9 [82]; comparison of viral shedding in ferrets of human seasonal and pandemic versus avian H5N1 viruses [83]	Focus on naturally viable variants; higher throughput; ability to test a wide range of phenotypes
Experimental production and evaluation in animal transmission models of reassortants or mutants of seasonal influenza to identify genetic components required for transmissibility, maintaining surface proteins to which human immunity exists	Replacing M segment of H3N2 and H1N1 strains with one from H1N1pdm to assess effect on guinea pig transmission [84]; ferret transmission assays of recombinant H1N2swine × H1N1pdm viruses to determine role of HA-NA balance [85]	Human transmissibility of parent viruses provide “natural” validation of animal model
Universal or broadly neutralizing influenza vaccine research	HA stalk vaccines [86],[87]; enhancing responses to conserved proteins [88]; T cell vaccination and improved adjuvants [89]; targeting universal NA epitopes [90]	Successful vaccine could eliminate need for rapid production of pandemic-specific vaccine and seasonal revaccination; complementary technology to other approaches
Studies of host factors using naturally occurring viruses	Identification of host factors restricting pathogenicity in animal models, in vitro, and via human genetics [91]	Potential therapeutic targets identified
Accelerating vaccine production	Sequence-based design and cell culture manufacture of influenza vaccine [92]	More rapid manufacture

HA, hemagglutinin; NA, neuraminidase.

More generally, it should be remembered that the public health goal is to curtail influenza pandemics and seasonal transmission [55],[56]. Exploring basic biology is just one scientific means to this end. Other approaches, such as developing universal influenza vaccines and novel antiviral drugs and strategies to enhance host responses, as well as improving technologies for rapid vaccine manufacture, are being pursued without risks of PPP release (Table 2).

## Paths Forward

We urge that proposals for any future experiments on PPPs be evaluated according to quantitative risk–benefit analysis guided by the principles of the Nuremberg Code. Indeed, HHS frameworks require a risk–benefit analysis to approve gain-of-function experimentation on H5N1 and H7N9 [33]–[35] viruses, yet no such analysis has been made public, if it has been conducted. Other funding and regulatory agencies, which have not yet called for a risk–benefit analysis, should require one as well. In biomedical grant review processes, proposals compete for limited funding, and most proposals that could advance science are never supported, because of budget constraints or because funding agencies conclude that there are more promising, safer, more humane, or otherwise superior ways to achieve scientific goals. PPP experimentation poses a significant risk to public health, arguably the highest level of risk posed by any biomedical research. Such experiments should be assessed on the basis of their marginal benefits, compared to those of safer approaches. In the case of influenza, given the higher throughput and lower cost of alternatives, we believe the benefits of alternative approaches will be greater than those of novel PPP experimentation, yet without the risks–thereby negating the justification for taking such risks. Similarly, careful consideration should be given to analyses of novel PPP experiments beyond the study of influenza, as these are proposed. Funders and regulators should evaluate the balance of risks and benefits before further novel PPP experiments are undertaken.

## Abbreviations

BSL: biosafety level
DURC: dual use research of concern
HHS: US Department of Health and Human Services
PPP: potential pandemic pathogen
